# Exhaustion of Skeletal Muscle Fibers Within Seconds: Incorporating Phosphate Kinetics Into a Hill-Type Model

**DOI:** 10.3389/fphys.2020.00306

**Published:** 2020-05-05

**Authors:** Robert Rockenfeller, Michael Günther, Norman Stutzig, Daniel F. B. Haeufle, Tobias Siebert, Syn Schmitt, Kay Leichsenring, Markus Böl, Thomas Götz

**Affiliations:** ^1^Mathematical Institute, University of Koblenz-Landau, Koblenz, Germany; ^2^Institute for Modelling and Simulation of Biomechanical Systems, Computational Biophysics and Biorobotics, University of Stuttgart, Stuttgart, Germany; ^3^Friedrich-Schiller-University, Jena, Germany; ^4^Department of Motion and Exercise Science, University of Stuttgart, Stuttgart, Germany; ^5^Hertie-Institute for Clinical Brain Research and Center for Integrative Neuroscience, Eberhard-Karls-University, Tübingen, Germany; ^6^Institute of Solid Mechanics, Technical University Braunschweig, Braunschweig, Germany

**Keywords:** fatigue, endurance time, parameter estimation, optimization, sensitivity analysis, biomechanics, first-order dynamics

## Abstract

Initiated by neural impulses and subsequent calcium release, skeletal muscle fibers contract (actively generate force) as a result of repetitive power strokes of acto-myosin cross-bridges. The energy required for performing these cross-bridge cycles is provided by the hydrolysis of adenosine triphosphate (ATP). The reaction products, adenosine diphosphate (ADP) and inorganic phosphate (P_*i*_), are then used—among other reactants, such as creatine phosphate—to refuel the ATP energy storage. However, similar to yeasts that perish at the hands of their own waste, the hydrolysis reaction products diminish the chemical potential of ATP and thus inhibit the muscle's force generation as their concentration rises. We suggest to use the term “exhaustion” for force reduction (fatigue) that is caused by combined P_*i*_ and ADP accumulation along with a possible reduction in ATP concentration. On the basis of bio-chemical kinetics, we present a model of muscle fiber exhaustion based on hydrolytic ATP-ADP-P_*i*_ dynamics, which are assumed to be length- and calcium activity-dependent. Written in terms of differential-algebraic equations, the new sub-model allows to enhance existing Hill-type excitation-contraction models in a straightforward way. Measured time courses of force decay during isometric contractions of rabbit *M. gastrocnemius* and *M. plantaris* were employed for model verification, with the finding that our suggested model enhancement proved eminently promising. We discuss implications of our model approach for enhancing muscle models in general, as well as a few aspects regarding the significance of phosphate kinetics as one contributor to muscle fatigue.

## 1. Introduction

Muscles performing mechanical work become exhausted, that is, they fail to maintain high force levels for a longer time period. What seems like the most obvious statement for everyone conducting physical exercises has not found its way into large parts of biomechanical modeling. Several state-of-the-art muscle models do not comprise the physiologically well-observed force decay over time, especially in fast-twitch fibers under high neural stimulation (Brown and Loeb, 2000; Rode et al., 2009; Blümel et al., 2012; Millard et al., 2013; Haeufle et al., 2014b; Schappacher-Tilp et al., 2015; Mörl et al., 2016). Moreover, literature terminology is vaguely summarizing any possible force-decay mechanism under the umbrella of *fatigue*, which is discussed throughout disciplines, such as biology (Enoka and Stuart, 1992; Fitts, 1994), biomechanics (Bergström and Hultman, 1985; MacIntosh et al., 2012), engineering (Böl, 2009), medicine (Chen et al., 1999; Cardozo et al., 2011), physiology (Lindström et al., 1977; Allen and Westerblad, 2001; Westerblad and Allen, 2002; Allen et al., 2008), and sports science (Komi, 2000; Brown et al., 2017). One of the first books addressing (muscle as well as whole body) fatigue was published at the beginning of the 20th century (Mosso, 1904) and a multitude of research has followed since, see Gandevia (2001) for a thorough review.

Commonly, muscle fatigue, i.e., the decline of the generable force level over time, is differentiated between *central fatigue*, i.e., the inability of the neural network to provide sufficient stimulation, and *peripheral fatigue*, i.e., the inability of the muscle cells to provide energy through metabolic activities (cf. Bigland and Lippold, 1954; Vøllestad, 1997). The main part (ca. 80%) of force decay is hereby associated with the latter, metabolic component (Kent-Braun, 1999). Due to the broad scientific interest, many metabolic factors of force decay have been identified, for example adenosine triphosphate (ATP) consumption (Sieck et al., 2013), calcium precipitation (Baylor and Hollingworth, 1998; Allen et al., 2008), glycolysis (Abbiss and Laursen, 2005), intracellular acidosis (Gandevia et al., 1995, Ch. 3), lactic acid (Tesch et al., 1978; Westerblad et al., 2002), pH value (Westerblad and Allen, 2002; Cooke, 2007), potassium loss or accumulation (Bickham, 2003; Allen et al., 2008) (Gandevia et al., 1995, Ch. 5), and reactive oxygen species (Debold et al., 2004; Cooke, 2007; Allen et al., 2008; Glass, 2017). A summarizing flowchart can be found in Abbiss and Laursen (2005, Figure 9). Although the role of some factors is controversial, e.g., the role of lactic acid (Tesch et al., 1978; Westerblad et al., 2002) or protons in general (Debold et al., 2016), it is agreed upon that the accumulation of inorganic phosphates (P_*i*_) accounts for the main factor in peripheral fatigue (Hibberd et al., 1985; Nosek et al., 1987; Kowaltowski et al., 1996; Allen and Westerblad, 2001; Westerblad et al., 2002; Debold et al., 2004). Despite vast physiological findings, biomechanical models, including force decay on the time scale of seconds, focus on rather phenomenological descriptions of typical force decay patterns (Liu et al., 2002; El ahrache et al., 2006; Xia and Frey-Law, 2008; Ma et al., 2009, 2012; Frey-Law et al., 2012; Rashedi and Nussbaum, 2015b), or try to cover the entirety of physiological processes (Shorten et al., 2007) which is computationally expensive.

Here, we develop a mathematically straightforward, yet physiology-based model that is able to explain the majority of early force decay while being computationally manageable. We introduce a model for hydrolytic ATP-ADP-P_*i*_ dynamics (or short *phosphate dynamics*) (Allen and Orchard, 1987) and the relative chemical potential ${\tilde{\mu }}_{\text{ATP}}$ of ATP as the corresponding state variable that is diminished by both ATP depletion as well as P_*i*_ accumulation. As validation, isometric contraction data at different muscle lengths from rabbit *M. gastrocnemius* and *M. plantaris* were used to reproduce typical force-time patterns. Our aim is to provide a basic enhancement of the well-known realm of Hill-type muscle models by describing bio-chemical kinetics that can be altered or extended, depending on the experimental resolution at hand.

## 2. Muscle Data

Experiments on female New Zealand white rabbits (*Oryctolagus cuniculus*, *n* = 2) with an average weight of 3.06 ± 0.01 kg (mean±SD) were approved according to section 8 of the German animal protection law (Tierschutzgesetz, BGBl. I 1972, 1277). The animals were anesthetized with Bupivacain (Jenapharm, 1 ml, 0.5%, epidural) after short-term sedation with sodium pentobarbital (Nembutal, 80 mg/kg body weight) and all efforts were made to minimize suffering. Anesthesia, preparation as well as experimental setup have been previously described (Böl et al., 2013, 2015; Siebert et al., 2015). In brief, the muscle to be examined was freed from its surrounding tissues and the rabbit was fixed with bilateral bone pins to a stereotaxic frame. The distal tendon of the muscle was attached horizontally to a muscle lever system (Aurora Scientific 310B-LR). Because the muscle performance depends on temperature, the animal was heated during the complete experimental procedure using a heating pad (Harvard Apparatus, 39.0 ± 0.4°C, mean±SD) and the surface of the isolated muscle was frequently sprinkled with heated (39°C) physiological saline solution.

For this study, we particularly investigated the performance of *M. gastrocnemius* (GAS) and *M. plantaris* (PLA). At the beginning of all measurements, the corresponding initial muscle lengths were determined *in situ* with a micrometer at an ankle and knee joint angle of 90°. Then a series of isometric experiments was performed at different muscle lengths (GAS: 114–120 and 128–132 mm; PLA: 114–124 mm; length increments of 2 mm) comprising the force-length relation from the beginning of its ascending limb (about 10% maximum isometric force, *F*_max_) to its plateau region (100% *F*_max_). To avoid muscle damage, the muscles were lengthened until passive forces reached about 10 and 20% of the maximum force for PLA and GAS, respectively. Thus, for GAS, there are two additional isometric measurements at the beginning of the descending limb of the force length relation (130 and 132 mm muscle length). Muscles were stimulated (Aurora Scientific 701C) with 100μs square wave pulses at 130 Hz (supramaximal tetanic muscle stimulation) via the tibial nerve using a bipolar gold electrode for 700 ms. One additional measurement for the GAS at 126 mm was conducted with 1,400 ms of stimulation.

## 3. Incorporating Bio-chemical Kinetics Into a Macroscopic Muscle Model

In this section, we work out an enhancement of an existing Hill-type muscle model (see Appendix A), which describes the dynamics of the *activation* and *contraction* of muscle fiber material. The model is enhanced by an additional process (*phosphate dynamics*), which is formulated in terms of a differential-algebraic equation.

Beforehand, in literature, we identified five distinguishable approaches of modeling short-term force decay patterns in skeletal muscle as a consequence of ongoing stimulation. Arranged with respect to increasing physiological verisimilitude these are

1. Functional effects (multi-parametric, control theoretical): introducing a muscle *compartment model*, whose states can be controlled to match desired data trajectories (Hawkins and Hull, 1993; Freund and Takala, 2001; Liu et al., 2002; Xia and Frey-Law, 2008; Böl, 2009; Frey-Law et al., 2012; Sih et al., 2012). For computational simplification, Wiener-Hammerstein models (Wiener, 1942; Narendra and Gallman, 1966; Wills et al., 2013) are also used (Cai et al., 2009).
2. Isolated functional effects (low-parametric): search for a functional dependency of force decay on calcium ions (Dorgan and O'Malley, 1998), calcium-troponin complexes (Ding et al., 2000; Marion et al., 2010), pH value (Giat et al., 1993), or glycolytic flux (Callahan et al., 2016) partly involving purely descriptive parameters bearing no relation to physiology.
3. Phenomenological details: introducing a theoretical construct, named for example *fatigue index* (Ma et al., 2009, 2011), *fitness level* (Tang et al., 2005), *fatigue rate* (Ma et al., 2012), *co-contraction factor* (Seth et al., 2016) or similar (Deeb et al., 1992; James and Green, 2012), which describes the empirically observed load-endurance relation per (linear) ODE. Reviews of applying this method can be found in (El ahrache et al., 2006; Ma et al., 2009; Rashedi and Nussbaum, 2015a,b).
4. Phenomenological chains: mathematically describing macroscopic processes based on measured aerobic and anaerobic power output (Eriksson et al., 2016).
5. Resolved physiological chains: mechanistically formulating interactions of bio-chemical processes with predictive power; potentially resulting in dozens of (partial) differential equations and more than 100 parameters (Shorten et al., 2007).

We aim at predicting the force decay of a biomechanical muscle computer model as response to a given muscle stimulus (forward dynamic simulation). Opposing, in approach (1) the force level is considered given and the stimulus is to be estimated in order to match the observed force decay (inverse dynamic simulation). Thus, here approach (1) can be ruled out. As well can approach (4), because it does not incorporate inner-muscular properties. An advantage of approaches (2) and (3) is the computational and epistemological simplicity, whereas a disadvantage is the missing physiological interpretability of the model parameters. In contrast, the physiological backbone of approach (5) is beyond dispute, however, so is its computational complexity. Our aim is to combine the strengths of approaches (2), (3), and (5), simultaneously neglecting their weaknesses, by developing a computationally cheap model based on physiological knowledge (cf. also section 5.1). As mentioned before as well as in Shorten et al. (2007), the accumulation of P_*i*_ as a consequence of ATP turnover accounts for the main reason for short-term force decay. Therefore, a single, linear ODE is added to the existing model (see Equation 3), describing the change in [ATP] and [P_*i*_] within the sarcoplasmatic reticulum and its interplay with cross-bridge mechanics, based on known reaction kinetics, for a detailed description see Appendix B.

Following the iconic paper of Lymn and Taylor (1971), ATP binds to the attached myosin head to allow its release from the actin filament. The energy release in the hydrolysis is then used to re-configure the myosin head for re-attachment in order to undergo another power stroke. Although many physiological details remain uncertain, the core element of the cross-bridge cycle can be represented by a simple pseudo-first order equilibrium

(1) $$\text{ATP}\overset{k_{\text{hyd}}:\text{ ATPase}+{\text{H}}_{2}\text{O}}{\underset{k_{\text{con}}:\text{ ATP Synthase}-{\text{H}}_{2}\text{O}}{⇌}}\text{ADP}+{\text{P}}_{i},$$

namely ATP hydrolysis/condensation. Herein, *k*_con_ and *k*_hyd_ denote the rates of condensation (catalyzed by mitochondrial ATP synthase) and hydrolysis (catalyzed by ATPase within the myosin head), respectively, with the corresponding equilibrium constant

(2) $$\begin{array}{l} K_{\text{ATP}}=\frac{k_{\text{con}}}{k_{\text{hyd}}}=\frac{\left[\text{ATP}\right]\cdot c_{0}}{\left[\text{ADP}\right]\cdot \left[{\text{P}}_{i}\right]}, \end{array}$$

where *c*_0_ = 1M represents the standard concentration serving as a normalization factor (Allen and Orchard, 1987; Hancock et al., 2005; Cooke, 2007). Following Equation (1), the time evolution of [ATP] or [P_*i*_] can be expressed in terms of the first order ODE

(3) $$\begin{array}{l} \frac{\text{d}}{\text{d}t}\left[{\text{P}}_{i}\right]=k_{\text{hyd}}\cdot \left[\text{ATP}\right]\cdot c_{0}-k_{\text{con}}\cdot \left[\text{ADP}\right]\cdot \left[{\text{P}}_{i}\right]. \end{array}$$

Note that although this dynamic is motivated by physiological considerations, it constitutes for a vastly oversimplified description of the bio-chemical processes underlying the full cross-bridge cycle. However, Equation (3) can be enhanced if explicit measurements (e.g., concentrations or time constants) of the involved reactants were available, see section 5.2 and in particular Figure 5.

For a start, in our model, *K*_ATP_ and in particular *k*_hyd_, are assumed to be dependent on the current amount of calcium-bound troponin C terminals $\tilde{q}$ as well as relative fiber length ${\tilde{\ell }}_{\text{CE}}$, cf. Appendix A. The condensation rate *k*_con_ is assumed to be a constant. The dependence of *k*_hyd_ on $\tilde{q}$ is obvious, because ATP hydrolysis within the myosin head does not occur if there are no available active sites on actin. It has been shown that, within resting muscle fibers, there is ATP consumption, but far less than in activated ones (Hilber et al., 2001, Figure 2A). This resting utilization corresponds to our model parameter ${\tilde{q}}_{\min}$ representing minimum troponin-activity. Hence, the rate constant *k*_hyd_ is assumed to increase along with $\tilde{q}$, i.e., to shift the reaction Equation (1) in favor of the hydrolysis products. Experiments further show that both ATP consumption rate (Aljure and Borrero, 1968; Doud and Walsh, 1995; MacNaughton and MacIntosh, 2007; Fenwick et al., 2016) and P_*i*_ release (Bickham et al., 2011, Figure 3a) are inversely proportional to fiber length (see also section 5.5). Summarizing, the following ansatz is obtained:

(4) $$\begin{array}{l} K_{\text{ATP}}=K_{\text{ATP}}\left(\tilde{q},{\tilde{\ell }}_{\text{CE}}\right)=\frac{k_{\text{con}}}{k_{\text{hyd}}\left(\tilde{q},{\tilde{\ell }}_{\text{CE}}\right)}=\frac{k_{\text{con}}}{{\hat{k}}_{\text{hyd}}\cdot \tilde{q}/{\tilde{\ell }}_{\text{CE}}}, \end{array}$$

with the parameter ${\hat{k}}_{\text{hyd}}$ representing the hydrolysis rate at full activity and optimal fiber length. Note that the alterations of the rate constants with respect to external conditions, such as fiber type, pH value, or temperature, are not (yet) incorporated (cf. section 5.3). It is further assumed that in a sufficiently activated fiber there is enough calcium to simultaneously enable the ATPase within the myosin head (De La Cruz and Ostap, 2009; MacIntosh et al., 2012; Glass, 2017). Note that magnesium, which serves a co-factor, is not yet incorporated within our model, and assumed to be sufficiently available.

Ultimately, two mechanisms of phosphate dynamics are incorporated

- ATP is to some extent refueled by phosphate storages, such as Creatinephosphate and ADP, cf. Equations (22)–(24) as well as Carlson and Wilkie (1974), Hilber et al. (2001), and
- a rising level of P_*i*_ (and simultaneously of ADP) deteriorates the relative chemical potential of ATP, denoted ${\tilde{\mu }}_{\text{ATP}}$, which is defined as
  (5) $$\begin{array}{l} {\tilde{\mu }}_{\text{ATP}}:=\frac{-\Delta G_{\text{ATP}}^{\circ }+R\cdot T\cdot \ln\left(\frac{\left[\text{ATP}\right]\cdot c_{0}}{\left[\text{ADP}\right]\cdot \left[{\text{P}}_{\text{i}}\right]}\right)}{\mu_{\text{ATP,max}}}, \end{array}$$
  where *R* denotes the universal gas constant, *T* the temperature, and μ_ATP, max_ ≈ 60 kJ/mol the maximum chemical potential of ATP in a resting fiber (Barclay, 2015) (see also Equations 37 and 38).

Incorporating the new state ${\tilde{\mu }}_{\text{ATP}}$ in our model, the term *muscle activity* (ã, cf. Appendix A) from now on refers to the relative amount of force-producing cross-bridges formed at the available active sites, i.e.,

(6) $$\begin{array}{l} \tilde{a}:=\tilde{q}\cdot {\tilde{\mu }}_{\text{ATP}}. \end{array}$$

Compare Appendix A.0.3 and Equation 21 for a quick assessment of the changes that arise in the underlying model from Günther et al. (2007).

Certainly, our introduced model enhancement is far from capturing every physiological cause of force decay in skeletal muscle. Yet, given that the full mechanisms of the remaining factors are at best partially known (MacIntosh and Rassier, 2002; Allen et al., 2008), it may arguably serve as a physiological and partly mechanistic basis for further enhancements. As the results in section 4 show, the addition of a single ODE containing only *one* new parameter ${\hat{k}}_{\text{hyd}}$ is already sufficient to model the short-term force decay in isometric contraction experiments. Table 1 summarizes the parameters necessary to describe the full phosphate kinetics as explicitly explained in Appendix B.

**Table 1:** Overview of the nine new model parameters, necessary to describe phosphate dynamics (cf. Appendix B).

**Symbol**	**Value**	**Unit**	**Source**	**Meaning**
[ADP]_0_	0.01	[mM]	Allen et al., 2008	Initial (*t* = 0) [ADP]
[ATP]_min_	1.2	[mM]	Allen et al., 2008	Minimum [ATP]
*c*_ad_	7	[mM]	Allen and Orchard, 1987	Total adenine concentration
*c*_cr_	25	[mM]	Allen and Orchard, 1987	Total creatine concentration
*c*_ph_	46	[mM]	Allen and Orchard, 1987	Total phosphate concentration
*K*_adk_	1	[ ]	Allen and Orchard, 1987	Adenylate kinase equilibrium constant
*K*_ck_	200	[]	Allen and Orchard, 1987	Creatine kinase equilibrium constant
*k*_con_	0.1	[Hz]	Linari et al., 2010	ATP condensation rate constant
${\hat{k}}_{\text{hyd}}$	Open	[Hz]	–	ATP hydrolysis rate constant

*All values for the eight fixed parameters were taken from literature. The optimized values for the open parameter ${\hat{k}}_{\text{hyd}}$ as well as the remaining model parameters can be found in Table 2*.

## 4. Results

**Figure 1:**
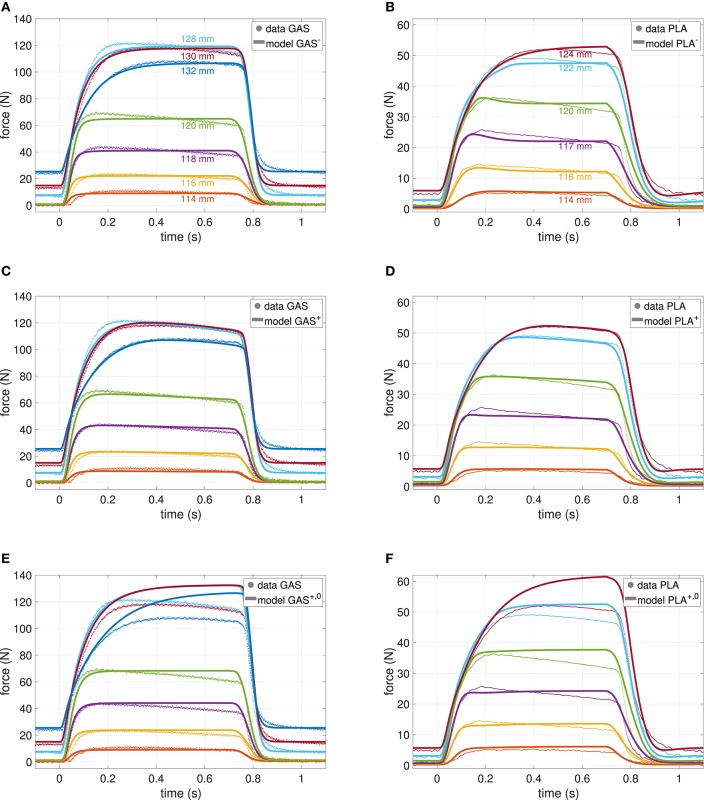
Force-time courses of GAS (left column) and PLA (right column) at various muscle lengths (see section 2). Models were fit to data, where different lengths are indicated by different colors as labeled in the top row. Parameter values are summarized in Table 2. **(A,B)** Classical model (GAS^−^, PLA^−^) without phosphate dynamics. **(C,D)** New model (GAS^+^, PLA^+^) including phosphate dynamics. **(E,F)** New model with switched off ATP hydrolysis (GAS^+, 0^, PLA^+, 0^, i.e., ${\hat{k}}_{\text{hyd}}=0$).

In total, *n*_GAS_ = 7 and *n*_PLA_ = 6 isometric force-time datasets from Siebert et al. (2015) for GAS and PLA, respectively, were compared with our model's output when applying the same stimulation protocol (control function) as within the experiments. Following Figure 1, each experiment had a runtime of 1.2 s, with a sampling rate of 1 kHz. The control function is zero, i.e., no electrical stimulation, for the first 0.1 s and is set to one, i.e., full stimulation, for further 0.7 s. Within the last 0.4 s, the muscle again experiences zero stimulation and finds its pre-stimulation equilibrium. It had been priorly shown that such isometric experiments are generally utilizable to estimate dynamic parameters (Rockenfeller and Günther, 2016), particularly within the force rise and fall phases at the beginning and the end of the stimulation. Valid estimations for static parameters, such as slack lengths and maximum force, are possible at the equilibrium states for zero and full stimulation, respectively.

In order to assess the effect of the new state introduced in Equations (3) and (37), as well as to increase validity, a comparative parameter estimation was conducted; once with the basic model as presented in Appendix A and once with the enhanced model. The formalization of the underlying optimization procedure can be found in Appendix C. Let GAS^−^ and PLA^−^ denote the models without phosphate dynamics, and let GAS^+^ and PLA^+^ indicate the inclusion. Further, we show the effect of switching off phosphate dynamics (${\hat{k}}_{\text{hyd}}=0$) in the inclusive model for which the nomenclature GAS^+, 0^ and PLA^+, 0^ was used. Table 2 lists the obtained parameter values as well as the pre-set boundaries (see next section 4.1). Figure 1 shows the associated force-time model outputs. These force-time results, obtained by least-square fits, are compared with respect to residues for both L_1_ (absolute) and L_2_ (squared) distances for the purpose of showing consistency throughout the model variants.

**Table 2:** Bounds and optimized parameter values resulting from the estimation process, described in Appendix C.

**Parameter**			**Model**		**Parameter**	**Model**	
**Symbol**	**Unit**	**Λ_*GAS*_bounds**	**GAS^−^**	**GAS^+^**	**Λ_*PLA*_bounds**	**PLA^−^**	**PLA^+^**
ℓ_SEE, 0_	[m]	[0.09…0.11]	0.0996	0.1017	[0.09…0.11]	0.1045	0.1054
Δ*U*_SEE, nll_	[]	[0.04…0.11]	0.103	0.0785	[0.04…0.11]	0.0620	0.0762
Δ*F*_SEE, 0_	[]	[80…160]	123	109	[50…70]	69.4	68.1
Δ*U*_SEE, l_	[]	[0.01…0.08]	0.0524	0.0482	[0.01…0.08]	0.0409	0.0414
$F_{\text{PEE}}$	[]	[0…1.2]	0.133	0.135	[0…1.2]	0.118	0.0908
$L_{\text{PEE}}$	[]	[0.9…1.5]	1.19	1.02	[1…1.5]	**1.00**	**1.00**
ν_PEE_	[]	[2.5…5]	**5.00**	3.16	[2.5…5]	**2.50**	**2.50**
Δ*W*_asc_	[]	[0.1…1]	0.215	0.404	[0.1…1]	0.237	0.268
Δ*W*_des_	[]	[0.1…1]	0.690	0.330	[0.1 …1]	0.429	0.570
ν_asc_	[]	[1.5…8]	4.10	6.75	[1.5…5]	**1.57**	1.84
ν_des_	[]	[1.5…6]	4.46	2.36	[1.5…5]	3.89	2.83
*F*_max_	[N]	[80…160]	120	133	[50…70]	57.1	63.7
ℓ_CE, opt_	[m]	[0.014…0.025]	0.0160	0.0198	[0.005…0.02]	0.0130	0.0106
*A*_rel, 0_	[]	[0.03…0.5]	0.0786	0.0655	[0.03…0.5]	0.244	0.240
*B*_rel, 0_	[1/s]	[1…10]	6.30	2.91	[1…10]	**10.0**	6.58
*D*_SDE_	[]	[0.1…10]	1.65	0.149	[0.1…10]	**10.0**	3.88
*R*_SDE_	[]	[0.01…1]	0.0233	**0.0100**	[0.1…1]	**1.00**	0.356
*S*_e_	[]	[1…4.5]	**1.00**	2.85	[1…4.5]	**4.50**	3.40
*F*_e_	[]	[1.1…2]	**1.10**	**1.14**	[1.1…2]	1.61	1.32
${\tilde{q}}_{\text{min}}$	[]	[0.001…0.01]	0.00576	0.00944	[0.001…0.01]	**0.0100**	**0.0100**
ϖ_opt_	[]	[1.5…10]	2.45	3.47	[1.5…10]	1.82	2.03
*m*	[1/s]	[5…20]	12.8	13.7	[5…20]	8.17	8.22
ν	[]	[2…8]	4.99	4.85	[2…8]	3.76	4.77
${\hat{k}}_{\text{hyd}}$	[1/s]	[1…3]	NaN	1.45	[1…3]	NaN	2.27

*Parameter values are given to three significant digits. The slack length of the serial elastic element constitutes for an exception with four digits due to the high sensitivity of the model with respect to this value (cf. Figure 3). Corresponding model output vs. data is shown in Figure 1. Parameter values within a 5%-neighborhood of the bound value are printed in bold*.

**Figure 2:**
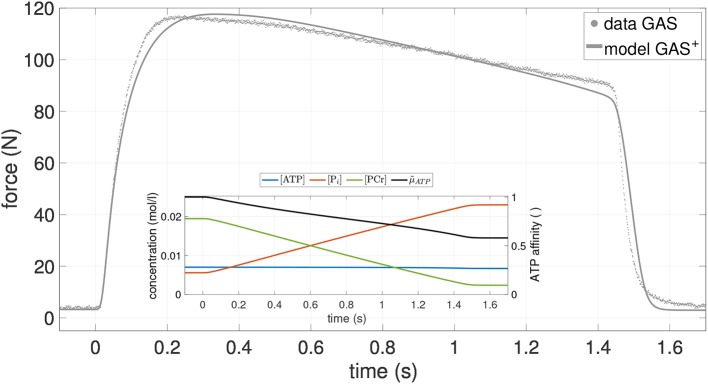
Force-time course of an additional isometric experiment on GAS (dots) as well as the model output of GAS^+^ (gray line), utilizing parameter values from Table 2. Stimulation duration is 1.4 s, GAS length ℓ_GAS_ = 126mm. Inlay shows the concentration-time courses of the most relevant bio-chemical reactants (blue: ATP; red: P_*i*_; green PCR) as well as the relative chemical potential of ATP (black: ${\tilde{\mu }}_{\text{ATP}}$). For more details see Figure A1 in Appendix B.

To further support the validity of the exhaustion model, we compared the model prediction with one additionally available experimental data set, namely an isometric contraction of GAS with longer stimulation time (1.4 s) at medium length (126 mm) (see Figure 2). In agreement with experiments, the model simulation yielded a decay in muscle force of 23% over the stimulation period. Thus, the model is able to predict isometric experiments with prolonged stimulation times. However, this statement only holds true for a single additional isometric contraction. Further long-stimulation experiments, also with PLA, should be conducted in order to strengthen this claim. As solely determined by isometric contractions, the herein obtained model parameters should in any case be cautiously considered when aiming at dynamic simulations with high shortening velocities.

### 4.1. Parameter Estimation

#### 4.1.1. Setting the Parameter Bounds

As presented in Appendix C, parameter estimation/optimization was conducted in a least-squares sense. It has been further possible to restrict the search area for the algorithm, i.e., pre-define a hyperrectangle in the parameter space by lower and upper bounds, see third and sixth column of Table 2. Most parameters settle well in between these pre-set bounds, which were chosen based on physiological plausible values and prior model knowledge (Mörl et al., 2012; Siebert et al., 2015; Rockenfeller and Günther, 2016). As far as possible, parameter bounds were given similar for GAS and PLA. Naturally, deviations in parameter values for these different muscles occurred in (slack) lengths as well as forces. Further, a challenge occurred due to the redundancy in serial and parallel elasticities. As is apparent from Equation (9), serial and parallel elastic element are assumed to operate in series. Further, MTU length is held constant (static) in isometric experiments. In consequence, the individual contributions of parallel and elastic elasticities of the muscle in dynamic situations can not be clearly resolved. Hence, restrictions in either parameter set might influence the convergence property of the other and the herein presented bounds have to be considered with caution.

#### 4.1.2. Comparison Between Model^−^ and Model^+^

The most prominent effect of including phosphate dynamics on parameter values is an about 10% increase of the parameter value of maximum isometric force (*F*_max_) as calculated by the optimization algorithm. The explanation of an increasing *F*_max_ is straight forward: The model^+^ has to compensate the force decay induced by phosphate accumulation. This effect becomes particularly visible in the terminally investigated model^+, 0^, where ATP hydrolysis and thus phosphate accumulation is artificially set to zero (see Figures 1E,F and next paragraph). Accordingly, the maximum velocity decreases ~40% in the model^+^, mostly due to a decreasing *B*_rel, 0_. Finally, serial damping is also less in the model^+^.

For the rest of the parameters, there is no systematic change detectable. Optimal fiber length increases from GAS^−^ to GAS^+^ but decreases from PLA^−^ to PLA^+^. The opposite holds true for the serial elastic element (SEE) parameters Δ*U*_SEE, nll_ and Δ*U*_SEE, l_. Activation dynamics parameters remain almost constant.

#### 4.1.3. Comparison Between Model^+^ and Model^+, 0^

When switching off ATP hydrolysis in the model^+^, thus inhibiting phosphate accumulation, model force continues to rise well above measured force levels, particularly at longer MTU length (cf. Figures 1E,F). This observation is in accordance with experiments (Phillips et al., 1993), where a decrease of inorganic phosphate was associated with an increase in force production (Westerblad and Allen, 2002). Hence, the value of *F*_max_ in the model^+^ now contains additional information about a theoretically achievable maximum force, if inorganic phosphate was pumped out of the muscle cells instead of accumulated.

#### 4.1.4. Comparison Between GAS and PLA Models

The optimized parameters are in good agreement with prior experiments (Siebert et al., 2015) (see also section 5.4). In only two (GAS), respectively one (PLA) experiment the CE reaches lengths above ℓ_CE, opt_ and forces close to *F*_max_. Hence, the shape of the descending branch of the force-length relationship as well as the results for the non-linear to linear transition of the SEE have generally to be taken with caution (cf. Figure 4).

In contrast to GAS^−^, the PLA^−^ model shows a more or less pronounced force overshoot after being fully stimulated, see particularly Figure 1B at medium lengths between 0.1 and 0.2 s. While trying to compensate for the apparent force decay, the optimizer found an interesting, non-trivial parameter setup: serial damping was adjusted to become very strong (*D*_SDE_ = 10) and completely force independent (*R*_SDE_ = 1), cf. (Günther et al., 2007, Equation 23). In return, the curvature of the eccentric part of the force-velocity relation was substantially increased (S_e_ = 4.5) in order to compensate a slow force decay at the end of the stimulation.

Maximum shortening velocity (*B*_rel, 0_/*A*_rel, 0_) is twice as large in GAS^−^ compared to PLA^−^, i.e., 80–44 ℓ_CE, opt_/s, and 50% larger for GAS^+^ compared to PLA^+^, i.e., 41–27 ℓ_CE, opt_/s. Mainly this difference is explained by *A*_rel, 0_ being 3.5 times larger for GAS than for PLA (see Table 2).

The force-length relation of GAS shows a broad plateau-like region with steep ascending and descending limbs, whereas PLA shows a shallow ascending limb (see Figure 4). We already mentioned the lack of trustfulness of the descending limb's shape. However, the difference in the force-length relation might additionally be influenced by pennation of the fibers. As the fiber shortens, its pennation angle increases (Drazan et al., 2019). At higher forces and lower velocities, the force-length characteristic of the fiber is then transformed (geared) to an altered force-length characteristic of the whole muscle (Azizi et al., 2008).

Finally, PLA (${\hat{k}}_{\text{hyd}}=2.27$) seems to metabolize ATP, and thus accumulate phosphate, more rapidly than GAS (${\hat{k}}_{\text{hyd}}=1.45$), which is in good agreement with the fiber type composition of PLA (> 90% fast twitch fibers) and GAS (> 75% fast twitch fibers) (Wang and Kernell, 2001; Siebert et al., 2015).

#### 4.1.5. Figures and Residues

Figure 1 shows the force-time measurements compared to the model evaluations for GAS (left column) and PLA (right column). Within these columns, the top row shows the corresponding best fit model^−^, the middle row the newly proposed model^+^ and the bottom row the effect of an a-posteriori neglect of exhaustion, i.e., model^+, 0^. The residues in the optimized least-square sense (L_2_) were for comparability scaled to 1000 data points and account for 61.43, 56.17, and 166.79 N for GAS^−^, GAS^+^, and GAS^+, 0^, as well as 32.19, 28.58, and 70.26 N for PLA^−^, PLA^+^, and PLA^+, 0^, respectively. Hence, fits for the model^+^ resulted in around 10% less error than for model^−^. Fits of GAS and PLA were comparably good with respect to the difference in maximum force, that is *F*_max_ was estimated twice as large for GAS than for PLA, and so were the residues. In order to give an alternative error estimate, independent of the objective function, the absolute (L_1_) deviation of model and measurement scaled to a single data point was calculated. These values are 1.57, 1.42, and 4.42 N for GAS^−^, GAS^+^, and GAS^+, 0^ as well as 0.84, 0.71, and 1.19 N for PLA^−^, PLA^+^, and PLA^+, 0^, respectively, therefore revealing the exact same qualitative behavior.

Summarizing, models^+^ yielded a superior fit, which additionally captured the force decay characteristic predicted in Equation (8), particularly for GAS^+^, i.e., small decay rate at short CE lengths increasing with length but becoming smaller again at CE lengths above ℓ_CE, opt_, see Figures 1C, 4. In Figure 1B the aforementioned effect of the strong damper in terms of an early force overshoot can be observed, especially for short lengths.

### 4.2. Parameter Sensitivity

**Figure 3:**
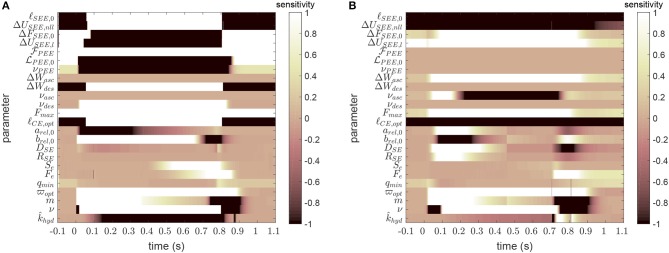
Example sensitivity-time matrices for each model parameter at a long length GAS (**A**, ℓ_GAS_ = 132mm, cf. dark blue curve in Figure 1A) and a short length PLA (**B**, ℓ_PLA_ = 116mm, cf. yellow curve in Figure 1B). Large absolute sensitivities indicate the local importance of parameter value accuracy. For further explanation see text. For scaling purpose, sensitivity values had been truncated at absolute values >1. For example for ℓ_SEE, 0_, sensitivities go up to absolute values in the magnitude of 10^4^.

Figure 3 shows the time courses of the sensitivity for all model parameters for two exemplary cases, one for a long GAS muscle and one for a short PLA. The (local) sensitivity values (see again Appendix C) indicate how small changes in the parameter value would affect the model output at the corresponding time instances. The absolute values were for comparability truncated at a value of one, which can be interpreted as for example a 10% change of the parameter value results in a 10% change in model output. Altogether, Figure 3 may help the reader to estimate the influence of parameters across time and magnitude as well as to acknowledge the setting of bounds in Table 2.

The model is most sensitive to reference and slack lengths of CE and SEE, respectively. On the one hand, they are properly determinable, but on the other hand they have to be known with high accuracy (cf. Table 2). For the longer muscle (Figure 3A), SEE, PEE and descending branch parameters gain importance, since the CE operates at lengths above ℓ_CE, opt_. Likewise, switches in sign of the sensitivities can be observed for all SEE and most PEE parameters, indicating the different influences in passive and active muscle states. For the shorter muscle (Figure 3B), parameters describing the ascending branch are of importance, as would have been expected. The influence of eccentric parameters is visible for the regions of force decay, be it due to phosphate accumulation or end of stimulation. The influence of Hill parameters on the model output is clearly visible after start and end of stimulation. Parameters for activation dynamics show similar behavior across MTU lengths; ${\tilde{q}}_{\text{min}}$ co-determines the passive force, whereas the remaining parameters begin to influence the model output right after the start and until the end of the stimulation.

### 4.3. Empirical and Theoretical Force Decay

**Figure 4:**
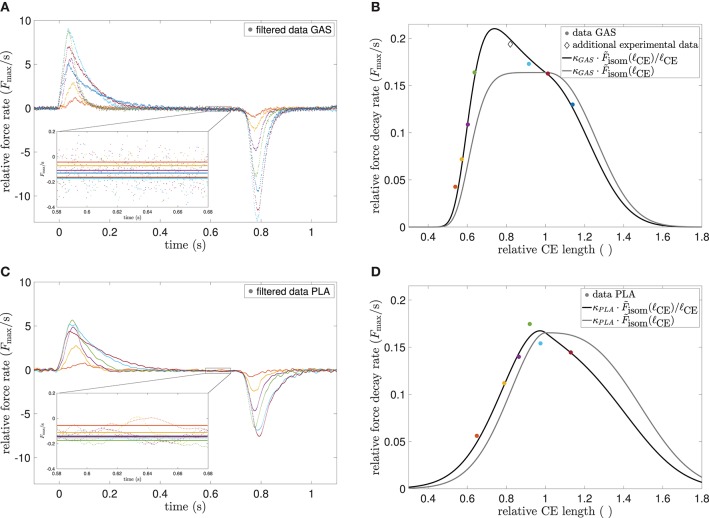
**(A)** Experimental force rates (time derivatives) of GAS at different MTU lengths, normalized to maximum force. Color encoding is the same as in Figure 1. Data were smoothed by a moving average filter with 40 ms width for clarity of depiction, but not for calculations. Inlay shows the mean force rates (solid lines) in the 0.1 s interval between 0.58 and 0.68 s, where near steady-state conditions were assumed. **(B)** Experimental force decay rates vs. CE length at different MTU lengths (colored circles) as extracted from **(A)** in comparison to theoretical modified CE force-length relation (Equation 8, black line). An additional data point (diamond) was taken from another GAS experiment (see Figure 2). The best-fit parameter κ_GAS_ = 0.163 s^−1^ was found by an optimization routine, all other parameters were taken from Table 2. The unmodified and scaled force-length relation [$\kappa_{\text{GAS}}\cdot {\tilde{F}}_{\text{isom}}\left(\ell_{\text{CE}}\right)$, gray line] is given for reference. In **(C,D)** the procedure is mirrored for PLA, where κ_PLA_ = 0.165 s^−1^.

As a direct consequence of Equations (6) and (21), the MTU force at the isometric steady-state ($\frac{\text{d}}{\text{d}t}\tilde{q}=\frac{\text{d}}{\text{d}t}\ell_{\text{CE}}=0$) can be written as

(7) $$\begin{array}{l} F_{\text{MTU}}{|}_{\frac{\text{d}}{\text{d}t}\tilde{q}=0,\frac{\text{d}}{\text{d}t}\ell_{\text{CE}}=0}=\tilde{q}\cdot {\tilde{\mu }}_{\text{ATP}}\cdot F_{\max}\cdot {\tilde{F}}_{\text{isom}}+F_{\text{PEE}}, \end{array}$$

which at full troponin-activity ($\tilde{q}=1$) results in a force decay rate of

(8) $$\begin{array}{l} \frac{\text{d}}{\text{d}t}F_{\text{MTU}}{|}_{\tilde{q}=1,\frac{\text{d}}{\text{d}t}\tilde{q}=0,\frac{\text{d}}{\text{d}t}\ell_{\text{CE}}=0}=\frac{\text{d}}{\text{d}t}{\tilde{\mu }}_{\text{ATP}}\cdot F_{\max}\cdot {\tilde{F}}_{\text{isom}}\sim {\tilde{F}}_{\text{isom}}/\ell_{\text{CE}}, \end{array}$$

see also Equations (37)–(39) for the latter proportionality. Indeed, the connection $\frac{\text{d}}{\text{d}t}F_{\text{MTU}}\sim {\tilde{F}}_{\text{isom}}/\ell_{\text{CE}}$ has, to our knowledge, not been formulated yet in the literature, but can be found in experimental data from rabbit psoas muscle (Hilber et al., 2001, Figure 2B) as well as in electromyography studies on center frequencies (Doud and Walsh, 1995, Figure 4). The former source omitted a functional dependency, whereas the latter gave a decreasing linear fit, although the shape of the altered sarcomere force-length relation was prominently shaped as described. Figure 4 shows the force rates of our data, compared to the theoretically predicted relation.

## 5. Discussion

### 5.1. Adding Submodels of Disregarded Physiological Processes

To predict the decay in muscle force during isometric contractions, we have added a (third) ODE (Equation 3), which describes the dynamics of [ATP], to an existing Hill-type muscle model which consisted of already two differential and several algebraic equations (see Appendix A). A. V. Hill's hyperbolic force-velocity relation (Hill, 1938), empirically found for muscle fibers, is in the core of Hill-type models. Therefore, such models are of empirical, macroscopic, and consequently reduced character. They often show deficiencies in their capabilities of reproducing the wealth of physiological and experimental conditions. For compensating one such deficiency, we have herein followed the methodological path of step-wise enhancing a Hill-type model. There are strong indications that the Hill relation, which has been inferred from the combination of mechanical and thermodynamic measurements, is founded in structural properties of muscle fibers (Günther and Schmitt, 2010; Rosenfeld, 2012; Seow, 2013; Rosenfeld and Günther, 2014; Günther et al., 2018), which also means that the Hill relation is not restricted to steady-state contractions (Piazzesi et al., 2002; Lemaire et al., 2016; Rockenfeller and Günther, 2016). Furthermore, because the Hill relation originates in both mechanics and thermodynamics, it may already well represent basic properties of active muscle tissue during *dynamic interactions* with other body tissues. We therefore conclude that the methodological path chosen has a sound basis.

**Figure 5:**
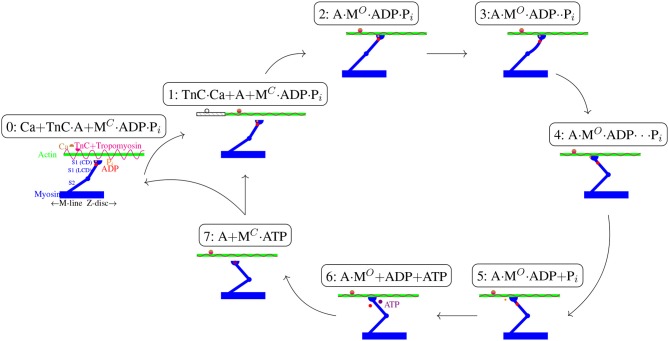
Schematic cross-bridge (Lymn-Taylor) cycle (1-2-3-4-5-6-7-1). New abbreviations are used as follows: Troponin C (TnC), actin (A), open and closed myosin switch-2-region (M^*O*^ and M^*C*^), light chain domain (LCD), catalytic domain (CD). For their explanations, see text. Although most processes are easily reversible, for the sake of clarity only one direction is depicted.

In this study, we have now enhanced the *activation dynamics* part of our initial model. In its initial formulation the model considered activation dynamics introduced by Hatze (1977, 1978, 1981) in a recently simplified variant (Rockenfeller and Günther, 2018). Hatze had thoroughly described the effect of a neural impulse on calcium dynamics up to the binding to troponin and subsequent clearance of tropomyosin from the actin helix (cf. processes P1 to P5 in Rockenfeller and Günther, 2016, Appendix A). The symbol $\tilde{q}$ for the troponin activity thus describes the relative amount of available (cleared) active sites on actin at a given filament overlap. However, he assumed that each possible cross-bridge would instantaneously form and generate force in the wake of *troponin activation*, thereby ignoring any further mechanisms and processes delaying or interfering with a cross-bridge's force generation. For example, as already motivated and sketched in Figure 5 in more detail, ATP hydrolysis reaction kinetics and phosphate accumulation are well-known mechanisms to have a bearing on cross-bridge dynamics. Therefore, we have introduced the ATP's chemical potential ${\tilde{\mu }}_{\text{ATP}}$ which is a transform of the additional state variable [ATP] that represents any force-reducing effects on single cross-bridges. This new state can likewise be interpreted as an attachment-to-detachment ratio in the sense of Huxley (1974).

It does not seem expedient to aim here for a higher (structural and parametric) resolution of the underlying processes within our model of activation dynamics, given and solely based on our experimental set-up (isometric whole muscle contractions). Yet, the chosen methodological path of step-wise model enhancement by adding structure-based equations is perfectly designed to do so, namely, to factor in, on the basis of experiments, processes like receptor-ligand binding processes and state transitions (Bagshaw and Trentham, 1973; Trentham et al., 1976; Stein et al., 1981; Grigorenko et al., 2007; Seow, 2013), further chemical reaction kinetics, and physical mechanisms like myosin head attachment (Nakajima et al., 1997) or ion diffusion, see the next sections 5.2 and 5.3. When trying to understand *which* process is effected by P_*i*_ kinetics, it is required to implement it in an even further enhanced model of muscle activation-contraction dynamics. The non-linear interactions of essential processes and mechanisms can only be understood in a cause-effect sense by mathematically formulating them in terms of at least algebraic but usually even differential equations and coupling these to state-of-the-art models formulated the same way. Eventually, this also applies to the very core of Hill-type models of muscle contraction: Hill's (phenomenological, steady-state) relation must then be replaced by a mechanistic model that represents both the basic force interactions and the thermodynamics on the cross-bridge level, formulated in terms of structure-based physical properties (cf. Günther et al., 2018).

### 5.2. Toward Resolving Characteristic Time Constants of Phosphate Dynamics

ODEs are used as standard method to model a characteristic evolution of an independent variable, which also depends on its own derivatives. Often, the time evolution of such an independent variable is studied in search for characteristic time constants of the modeled systems. The main Equation (3) of this contribution represents the time evolution of [ATP] or [P_*i*_] with the characteristic time constants for condensation *k*_con_ and hydrolysis *k*_hyd_. This ansatz together with the introduction of the state ${\tilde{\mu }}_{\text{ATP}}$ leads to the observed force decay $\frac{\text{d}}{\text{d}t}F_{MTU}$ (cf. Figures 1, 4). As described in the methods (section 3), the constants themselves describe a system characteristic, which represents a snapshot of even more detailed underlying processes.

Typical underlying processes for ATP hydrolysis *k*_hyd_ are the filling and draining of the ATP reservoirs including the actual type of flow during filling and draining: laminar or turbulent, the mixing of materials within the reservoir, and the residence time of the ATP on the myosin, i.e., the time ATP spends at the myosin head. Additionally, recent discoveries hint at two different types of ATP-myosin binding (Amrute-Nayak et al., 2014). The authors proposed that each myosin head has one site for ATP switching between two conformers (Tesi et al., 2017), which affects the binding duration and thus the characteristic time constant in the ODEs.

Admittedly, our model provides a rather phenomenological description of phosphate-induced force decay. In our activation model enhancement, the parameter ${\hat{k}}_{\text{hyd}}$ determines the corresponding characteristic time of decay. It is remarkable that ${\hat{k}}_{\text{hyd}}$ was the *only* free parameter in the enhancement part, that is, just its value was left to the parameter estimation process, whereas the other eight parameter values (cf. Table 1), were a priori fixed according to literature. The characteristic time 1/${\hat{k}}_{\text{hyd}}$ of force decay is of the order 0.5 s. Force *decay* along with phosphate *accumulation* in the sarcoplasma is surely the physiological process that has a functional effect in natural contraction conditions. Yet, phosphate *dilution* can have the reciprocal impact of *increasing* isometric force (Phillips et al., 1993; Tesi et al., 2002). We suggest therefore to neutrally term the model enhancement “phosphate dynamics” rather than anything like “phosphate fatigue” or “phosphate-induced force decay.” The phosphate dynamics examined here is slow as compared to all other processes in the cross-bridge (Lymn-Taylor) cycle. Phosphate dynamics is thus rather a boundary condition for the Lymn-Taylor cycle than strongly and non-linearly interacting with cross-bridge dynamics. Therefore, the choice of our methodological path (see section 5.1) is a non-critical issue for our findings.

Other chemical factors that are known to have an effect on cross-bridge cycling, such as magnesium contributions, calcium precipitation, or glycolysis, have so far not been incorporated into our enhanced model of activation dynamics. Accordingly, the narrowing down to adding a single ODE for [ATP] dynamics seems to be nothing else than mathematically formulating the Lymn-Taylor cycle (Lymn and Taylor, 1971). Figure 5 gives a schematic overview of this cycle in accordance with our current interpretations (cf. processes P6 to P8 in Rockenfeller and Günther, 2016, Appendix A). Additionally, we included the illustration of a recent mechanistic idea of how the power stroke may be driven by Coulomb repulsion of ADP^2−^ and ${\text{P}}_{i}^{2-}$ (Rosenfeld, 2012; Günther et al., 2018). Our scheme is further consistent with crystallography measurements of geometric configurations (conformational states) of the myosin S1 part (Geeves and Holmes, 1999, pp. 700ff):

0: Without calcium available, tropomyosin blocks the access of myosin to new actin sites. Hence, myosin heads might be bound in a rigor formation, cf. state 6, or detached with either ATP (state 7) or its hydrolysis products present in the catalytic domain of the S1 region. In the former case, the switch-2 region of myosin is in an OPEN state, which facilitates phosphate release; in the latter case, the switch-2 region is CLOSED, i.e., forms some kind of phosphate-“pocket,” which enables ATP hydrolysis (Geeves and Holmes, 1999).
1: Soon after a calcium ion has bound to a troponin C terminal, several active sites on actin are exposed, where the exact number as well as the underlying mechanism are yet controversial, (Reconditi, 2006; Deasi et al., 2015). The gray continuation to the left of the actin filament accounts for the post-power-stroke translation after one full cycle (1-…-7), i.e., re-entering step 1 from step 7.
2: The catalytic domain binds to an available active site and changes its state from CLOSED to OPEN.
3: According to Elliott and Worthington (1994), Lampinen and Noponen (2005), and Rosenfeld (2012), the basic force that drives the power stroke is of electro-static (Coulomb) character: the OPEN state then allows the negatively charged phosphate to push away from the likewise negative charged ADP, and the Coulomb force is levered by the light chain domain to cause elastical deformations (bending) in the light chain domain itself as well as in myosin and actin filaments. All of these deformations summing up to ~4 nm pre-strain (Piazzesi and Lombardi, 1995; Piazzesi et al., 2002) in the isometric condition.
4: Eventually, as P_*i*_ and ADP move further away from each other, the actual power stroke (including release of the elastically stored energy) causes the S1 region to rotate relative to the S2 region and consequently moves the myosin rod by an equivalent of another about 7 nm (Günther et al., 2018, Figure 2) at its tip (attachment point to actin), with the whole power stroke length being altogether about 11 nm (Piazzesi and Lombardi, 1995; Piazzesi et al., 2002).
5: *After* having transformed the chemical free energy to mechanical work, phosphate is finally released to the sarcolemma (Takagi et al., 2004; Muretta et al., 2015). This process might be slowed down or hindered by higher [P_*i*_] in the surrounding (Tesi et al., 2002).
6: In a consecutive step, ADP is likewise released into the sarcolemma (Suzuki et al., 1998), possibly with the aid of magnesium (Geeves and Holmes, 1999), leaving the actomyosin complex in a rigor formation.
7: In the presence of ATP, the myosin head detaches, switching again from OPEN to CLOSED state, allowing for another hydrolysis and the beginning of a new cycle. Just as step 5, this process can also be interfered with by a higher [P_*i*_] (Kerrick and Xu, 2004). Note that ATP hydrolysis is commonly assumed to take place before re-attachment, but might also take place afterwards (Tonomura et al., 1966; Adelstein, 1980).

In all, the presented model (Equation 3) subsumes several even more detailed processes by assuming *k*_hyd_ and *k*_con_. It remains open to integrate these into the equation, additionally. As was shown in this contribution, prerequisites are data of very good quality and a first guess of the system dynamics. Until then, the presented approach integrates phenomenologically-based molecular kinetics into macroscopic muscle models, enhancing them tremendously.

### 5.3. On the Effects of Phosphate Accumulation and Myofibrillar Calcium Sensitivity

The process of fatigue is commonly divided in three phases: An early decay of force, a plateau phase, and a late decay of force (Lännergren and Westerblad, 1991; Westerblad and Allen, 1991). The presented model considers the simulation of the early phase of fatigue. As the main cause for early force decay, single fiber experiments revealed (1) a decreased ability of the actomyosin crossbridges to generate force and (2) reduced myofibrillar [Ca^2+^] sensitivity (Allen et al., 2008). The first component is associated with elevated [P_*i*_] due to the breakdown of PCr. When [P_*i*_] had been elevated from below 1 to ~14 mM, a force decrease of about 50% was observed in [Ca^2+^]-activated skinned fiber experiments (Millar and Homsher, 1990, Figure 2). However, early skinned fiber experiments were conducted at non-physiological temperatures (10 to 15°C). The effect of elevated [P_*i*_] on force might decrease with increasing temperature to physiological relevant conditions (Debold et al., 2004; Ranatunga, 2010). Nocella et al. (2017) examined the effect of [P_*i*_] on the cross-bridge kinetics at physiological temperatures (33°C) and observed a fiber force decrease of 30% after 25 tetanic stimuli (on:off cycle = 0.4:1.5 s). Focusing on the time course of force decay (Nocella et al., 2017, Figure 2) after two tetanic stimuli (0.8 ms stimulation time, i.e., comparable to our presented experiments) they observed a force decrease of about 5%, which is similar to our observations (cf. Figure 1). Furthermore, they showed that the decrease in tetanic force mainly results from depressing the individual cross-bridge force and accelerated cross-bridge kinetics. However, force reduction during the early phase of fatigue was also associated with reduced myofibrillar [Ca^2+^] sensitivity (Debold, 2016). It was shown that the force decayed by 10% while the myoplasmic [Ca^2+^] increased (Westerblad and Allen, 1991). This increase is interpreted as the result of reduced myoplasmic [Ca^2+^] buffering (Westerblad and Allen, 1993). Alterations of the myofibrillar [Ca^2+^] sensitivity might occur due to increase of elevated metabolites as [H^+^] and [P_*i*_]. Nelson and Fitts (2014) observed at a pH of 6.2 in skinned muscle fibers (at 30°C) a decreased myofibrillar [Ca^2+^] sensitivity. The myofibrillar [Ca^2+^] sensitivity decreased even more when they added 30 mM [P_*i*_]. It was concluded that both low pH and elevated [P_*i*_] have a substantial effect on myofibrillar [Ca^2+^] sensitivity. However, experiments with intact single fibers show a minor effect of acidose on tetanic force decrease (<10%) (Westerblad et al., 1997). The role of acidosis in acute fatigue remains controversial and a major unresolved issue is whether the force-reducing effects of elevated [P_*i*_] in fatigue are amplified by the concomitant acidosis (Cheng et al., 2018). In our experiments, we can almost exclude the possibility that the pH decreased to values of 6.2 within a time period of 0.8 s (Stutzig et al., 2017). Thus, it seems that the main cause of fatigue that we observed and simulated is based on elevated [P_*i*_], which influences both actomyosin cross-bridge force generation and myofibrillar [Ca^2+^] sensitivity.

### 5.4. Comparison of Muscle Parameters

Muscle parameters of the present study were determined by fitting the muscle model to a series of isometric contractions (*n* = 6…7). Typically a much higher number of experiments (*n* = 20…40, Scott et al., 1996; Curtin et al., 1998; Wagner et al., 2005; Siebert et al., 2008) must be used to determine these muscle model parameters, including for example isometric, isotonic, isokinetic, and quick-release experiments. Thus, from an experimental point of view, the herein applied model-based parameter estimation (fitting method) (Wagner et al., 2005; Siebert et al., 2007; Rockenfeller and Günther, 2016) is more efficient compared to the classic procedure. Rabbit GAS and PLA muscle parameters have been previously determined with classic methods (Siebert et al., 2015). In general, their results are in good agreement with muscle model parameters determined in the present study. Maximum shortening velocity was overestimated (GAS: factor 1.7; PLA: factor 3.1) compared to classic methods, but consistent with recent findings on non-steady-state contractions (Piazzesi et al., 2002; Rockenfeller and Günther, 2016; Günther et al., 2018). Furthermore, maximum power deviates by 25% compared to classic methods. This can be explained by the limited parameter range available in the fitting method. Shortening velocity of the contractile component reaches maximally 0.5 *v*_max_ in isometric contractions used for parameter fitting. Thus, uncertainty in parameter estimation is higher for *v*_max_ and *P*_max_ compared to classic methods, in which *v*_max_ can be approximated by contractions against low loads or unloaded contractions (Edman, 1979). In contrast, parameters of the force-length relation are almost similar between classic and fitting method. There were differences in optimum muscle length (GAS: 11%; PLA: 20%) and widths of the ascending limb (GAS: 2%, PLA: 17%). For the SEE characteristic, our fit yielded a rather broad non-linear toe region (Δ*U*_SEE, l_ ≈ 8 vs. 4.9% for GAS and 3.6% for PLA with classic methods), with a transition to the linear region at around *F*_max_ and a less stiff behavior in the linear region (*K*_*SEE, l*_ ≈ 22.4 kN/m Günther et al., 2007, Equation 4 vs. *k* ≈ 30.3 kN/m Siebert et al., 2015, Equation 5 for GAS and *K*_*SEE, l*_ ≈ 15.6 kN/m vs. *k* ≈ 21.9 kN/m for PLA). Determination of PEE characteristics is limited to the passive force range (GAS: 20% *F*_max_; PLA: 9% *F*_max_) covered by isometric experiments at different muscle lengths (see Figure 1). Differences in PEE stiffness at longest muscle length is 10% for GAS and 25% for PLA. Deviations in fitted compared to experimental (classic method) PEE stiffness of PLA occurred due to lower passive force range available for parameter fitting (compared to GAS) as well as comparably low PEE forces (<5 N) and thus small impact on model simulations. Thus, there are only small differences in PEE forces (equaling passive force in the inactive muscle) between experiment and model simulation, see Figure 1 at *t* < 0.

### 5.5. Length-Dependence of Fatigue

**Figure 6:**
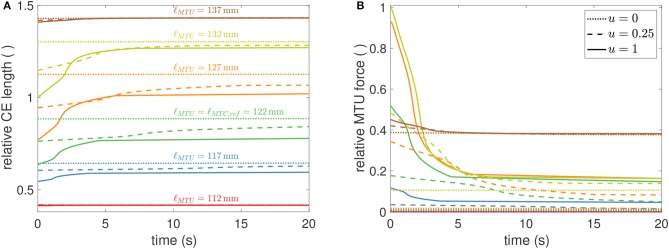
Simulated time courses for CE length **(A)** and MTU force **(B)** for a GAS muscle during isometric contractions, utilizing fixed parameters from Table 2. CE length are displayed relative to ℓ_CE, opt_ ≈ 0.0198m and forces relative to *F*_max_ ≈ 133N. Muscle length varies about −10, …, +15mm, in steps of 5 mm, around the reference length ℓ_MTU_ = 122mm. Colors and line-styles in both sub-figures are coherent, indicating MTU lengths [as specified in **(A)**] and stimulation levels [as specified in **(B)**]. Two observations should be highlighted. First, the force level in **(B)** at which (theoretically) infinite exhaustion time occurs settles at around 0.16, corresponding to 16%*F*_max_, which is in perfect agreement with the 15%*F*_max_ from *in vivo* experimental data (Rohmert, 1960). Second, the force at the longest muscle length [blue line in **(B)**] settles at significantly higher values, which is due to passive (PEE) forces that were herein not modeled to show any exhaustion.

Muscular fatigue plays an important role in the assessment of work-place ergonomics in order to accurately predict demands on workers with respect to the muscular forces required for their work tasks. To this end, endurance time is a measure used to characterize muscular loading situations. It was introduced to quantify the time a subject can hold a specific load by muscular contraction (Rohmert, 1960) and has been used to study many different postures and muscles (e.g., Frey-Law and Avin, 2010). In general, measurements reveal that endurance time is shorter for higher muscular forces. Furthermore, experiments show that below a certain load, endurance time becomes very long indicating a muscular load where the normal ATP resynthesis rate is sufficient to compensate the static energy requirement for the muscles. This lower threshold may be somewhere between 2 and 20% of the maximum voluntary contraction (van Dieën and oude Vrielink, 1994; El ahrache et al., 2006). The model presented here also shows this behavior (Figure 6), although we prefer the term *exhaustion time* when talking about the inability to maintain a certain, length-dependent force. Herein, exhaustion time was defined to be the first time instant where force had decayed more than 5% of its initial value at *t* = 0 s. For stimulation values around 0.1, exhaustion time may rise well above 20 s.

**Figure 7:**
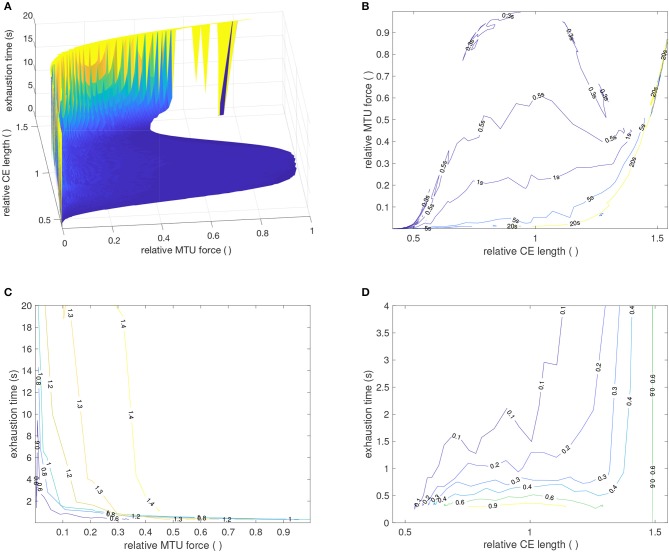
**(A)** Simulated force-length-exhaustion time diagram for variations in MTU lengths and stimulation levels as given in Figure 6. **(B–D)** Contour plots through the three different planes in **(A)**. In **(B)**, the force-length diagram for various exhaustion times is shown. The active and passive force-length characteristic of the CE is clearly visible. In **(C)**, the force-exhaustion time courses for various CE length are displayed. The longer the CE, the longer the exhaustion time. The typical exponential (or hyperbolic) characteristic (Rohmert, 1960; Frey-Law and Avin, 2010) is visible. **(D)** Shows CE length-exhaustion time curves for various force levels. The higher the force, the shorter the exhaustion time at the same length. For longer muscles, the passive force determines the exhaustion time.

In addition, the model predicts a length dependence of the exhaustion time (Figure 7). One core assumption of the model is that the hydrolysis rate decreases with increasing muscle fiber length (see Equation 4). This assumption was derived from the experimental observation that the force decay due to exhaustion scales with muscle length, see experimental data in Figure 4. This characteristic is, thus, immediately reproduced by the model, as shown in Figure 4 and Equation (8). More precisely, our model strongly suggests that the mechanism(s) that govern the exhaustion process are the same as those governing the length dependency of ATP hydrolysis.

This is an issue also discussed in the literature with respect to endurance time. Interestingly, the findings are ambiguous. Some experiments also found that fatiguability is reduced for longer muscle lengths, i.e., endurance time increases with muscle length (in accordance with our model, cf. Figure 7D) (Sacco et al., 1985; de Haan et al., 1986; Matthijsse et al., 1987; McKenzie and Gandevia, 1987; Arendt-Nielsen et al., 1992; Kawakami et al., 2000; Rassier, 2000). However, a few experiments also indicate that endurance time is reduced for increasing muscle length (Fitch and McComas, 1985; Ng et al., 1985; Willems and Stauber, 2002; Kooistra et al., 2005; Lee et al., 2007). This would be in contrast to our model prediction. In many of these studies, however, only few lengths were investigated omitting a direct conclusion on the length dependence. Finally, the data shown in Petrofsky and Phillips (1980) for endurance time in relation to elbow angle indicates that there is a maximum endurance time for an elbow angle of 120°, which is also the elbow angle for which the subjects strength is maximal. This indicates, that the endurance time may be related to the force-length relation of the muscle, an effect also visible in our model.

There is one important difference though between *in-vivo* endurance time in human workers and the exhaustion time predicted by our model: While muscular fatigue in humans may (partially) be compensated for by increasing motor-unit recruitment, i.e., increased muscle stimulation, we here simulate exhaustion under constant muscle stimulation input (*u* = 1). This additional recruitment is crucial for the notion of endurance time (Petrofsky, 1978) and can be seen in humans using muscle surface electromyograms (EMG) (Gamet and Maton, 1989; Maton and Gamet, 1989). It is actually used as an indicator of fatigue in ergonomics (Jørgensen, 1997; Nussbaum et al., 2001; Garg et al., 2002). While this aspect has to be considered in ergonomics, our model approach, for the first time, allows a systematic model-based analysis of the length-dependence of muscular exhaustion.

## 6. Summary

In this work, we developed a model able to explain the time course of force decay, which occurs as a consequence of ongoing neural stimulation. As opposed to the widespread but rather diffuse term *fatigue*, we here prefer to term the muscle's incapability of maintaining a certain force level as *exhaustion* in case the dominating mechanism behind fatigue is a shift in the equilibrium of the ATP hydrolysis-condensation kinetics (*phosphate dynamics*). Accordingly, we assume as the key feature of exhaustion the deteriorating chemical potential, i.e. — the change in internal energy per change in particle number — of ATP during hydrolysis. More precisely, decreasing [ATP] as well as increasing either [ADP] or [P_*i*_] yields a lower chemical potential being reflected in exhaustion. We incorporated phosphate dynamics into an established Hill-type muscle model representing excitation-contraction dynamics. This new (merged and enhanced) model was validated by parameter estimation with using experimental data of isometric contractions gathered from two types of rabbit calf muscles. With parameter values obtained from optimally fitting direct dynamic model simulations to experimental contraction data, the model can reproduce experimental findings strikingly better than the initial model. Moreover, the parameter values well agree with prior estimates from literature and eventually allow for predicting measurements from experiments with longer stimulation duration. We argue that the presented methodology of model enhancement can and ought to be applied to further physiological mechanisms, e.g., the Lymn-Taylor cycle or the impact of changes in myofibrillar calcium concentration. Lastly, consequences of the length-dependencies within the model have been investigated and linked to known findings from ergonomics.

## Data Availability Statement

All datasets generated for this study are included in the article/supplementary material.

## Ethics Statement

Experiments were approved according to section 8 German animal protection law (Tierschutzgesetz, BGBlI 1972, 1277) by the competent authority for animal welfare in Thuringia, Germany (Landesamt fur Verbraucherschutz, Abteilung Gesundheitlicher und technischer Verbraucherschutz; Permit Number: 02-022/11 and 02-027/14).

## Author Contributions

RR developed the model idea as well as the first draft of the manuscript and coordinated the partners. MG contributed the physiological insights, particularly during the parameter optimization and within section 5.1. DH investigated the aspect of length-dependency within the model as well as the inter-linkage with ergonomics, particularly in section 5.5. NS discussed the biological ramifications (cf. section 5.3) thus assuring to keep the model as straightforward as possible. TS, KL, and MB provided the data. TS further assessed the optimized parameter values (see section 5.4). SS provided the valuable discussions, final revision, and co-authored section 5.2. KL conducted the experiments and supervised the methodological aspects. MB conducted the several discussions as well as a final revision. TG helped in analyzing the mathematical methods (see the Appendices).

## Conflict of Interest

The authors declare that the research was conducted in the absence of any commercial or financial relationships that could be construed as a potential conflict of interest.

## Figures and Tables

**Figure A1:**
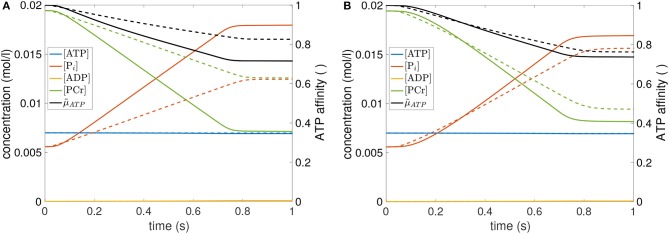
Time courses of [ATP], [P_*i*_], [ADP], and [PCr] (left ordinate) as well as ${\tilde{\mu }}_{\text{ATP}}$ (right ordinate) for GAS **(A)** and PLA **(B)**. Solid lines represent the time courses for the shortest muscle examined in our experiments (cf. **Figure** **1**). Dashed lines represent the respective longest muscle. Time courses for [Cr] are not shown due to a near congruence with [P_*i*_](*t*). Note that the ATP affinity decreases substantially, due to increasing P_*i*_, while the [ATP] itself remains almost constant.

## Appendices

### A. Initial muscle model

#### A.0.1. Model nomenclature

The herein sketched macroscopic Hill-type muscle model is completely based on prior publications (Günther et al., 2007; Haeufle et al., 2014b; Mörl et al., 2012), see also (Rockenfeller and Günther, 2016, App. A). Let ℓ_*X*_ and *F*_*X*_ denote the length and force, respectively, of element *X*, which represents either the whole muscle tendon unit (MTU), the fiber material (contractile element, CE), the connective tissue (parallel elastic element, PEE), or the tendon (serial elastic element, SEE, and serial damping element, SDE).

#### A.0.2. Model assumptions

It holds that ℓ_CE_ = ℓ_PEE_, as well as ℓ_SEE_ = ℓ_SDE_, and ℓ_CE_+ℓ_SEE_ = ℓ_MTU_. Further, the force equilibrium

(9) $$\begin{array}{l} F_{\text{MTU}}=F_{\text{CE}}\left(\tilde{q},\ell_{\text{CE}},\frac{\text{d}}{\text{d}t}\ell_{\text{CE}}\right)+F_{\text{PEE}}\left(\ell_{\text{CE}}\right)=F_{\text{SEE}}\left(\ell_{\text{CE}},\ell_{\text{MTU}}\right)\\ \;+F_{\text{SDE}}\left(\tilde{q},\ell_{\text{CE}},\frac{\text{d}}{\text{d}t}\ell_{\text{CE}},\frac{\text{d}}{\text{d}t}\ell_{\text{MTU}}\right) \end{array}$$

is assumed to hold at any time instance, see (Haeufle et al., 2014b, Figure 1) for a model sketch.

#### A.0.3. Activation dynamics

After a piece-wise continuous neural impulse *u* = *u*(*t*) at the neuromuscular junction, the relative calcium ion concentration $\tilde{c}$ within the sarcolemma changes with a time constant *m* via the ODE

(10) $$\begin{array}{l} \frac{\text{d}}{\text{d}t}\tilde{c}=m\cdot \left(u-\tilde{c}\right). \end{array}$$

Subsequently, calcium binds to troponin C and causes tropomyosin to move aside from the actin filament, thereby uncovering the seven active sites (Reconditi, 2006). The relative amount of calcium-bound troponin is commonly referred to as (troponin-)*activity* (Hatze, 1977, 1978, 1981) and denoted $\tilde{q}$. This process, herein referred to by the term *activation dynamics*, is known to be length-dependent (Kistemaker et al., 2005):

(11) $$\begin{array}{l} \tilde{q}\left(\tilde{c},{\tilde{\ell }}_{\text{CE}}\right)=\frac{{\tilde{q}}_{\min}+{\left(\varpi \left({\tilde{\ell }}_{\text{CE}}\right)\cdot \tilde{c}\right)}^{\nu }}{1+{\left(\varpi \left({\tilde{\ell }}_{\text{CE}}\right)\cdot \tilde{c}\right)}^{\nu }},\text{ where }\varpi \left({\tilde{\ell }}_{\text{CE}}\right)=\varpi_{\text{opt}}\cdot {\tilde{\ell }}_{\text{CE}}. \end{array}$$

The formulation includes a minimum troponin-activity ${\tilde{q}}_{\min}$ of the muscle at rest and a linear length dependency function ϖ describing the troponin C density with varying interfilamentary spacing (Rockenfeller and Günther, 2018), based on sarcomere volume constancy (Dragomir, 1970). The Hatze exponent ν has previously (Rockenfeller and Günther, 2017a) been equated to the exponent of the Hill *equation* (Hill, 1910). However, so far no mechanistic explanation has been provided on how to convert the relative amount of calcium-bound troponin (troponin-activity: $\tilde{q}$) to the relative amount of built-up cross-bridges (actual activity: ã), i.e., it is implied that myosin heads will automatically bind to available active sites and proceed with force generation. In section 3, we investigated this missing link of cross-bridge formation from a physiological point of view and incorporate the concept of *phosphate dynamics*. For this summary, the symbol ã is used for overall activity and the reader may keep in mind that it incorporates both, activation and phosphate dynamics.

#### A.0.4. Force-length-velocity relation

Depending on filamentary overlap, the muscle fibers are assumed to produce a force proportional to the isometric force-length relation (FLR), cf. (Günther et al., 2007; Rockenfeller and Günther, 2017b):

(12) $${\tilde{F}}_{\text{isom}}({\tilde{\ell }}_{\text{CE}})=\{\begin{array}{ll} \exp\left(-{\left|\frac{{\tilde{\ell }}_{\text{CE}}-1}{\Delta W_{\text{asc}}}\right|}^{v_{\text{asc}}}\right), & \text{if }{\tilde{\ell }}_{\text{CE}}\leq 1\\ \exp\left(-{\left|\frac{{\tilde{\ell }}_{\text{CE}}-1}{\Delta W_{\text{des}}}\right|}^{v_{\text{des}}}\right), & \text{if }{\tilde{\ell }}_{\text{CE}}>1 \end{array}.$$

The parameters Δ*W*_asc_, Δ*W*_des_, and ν_asc_, ν_des_ represent the width and kurtosis of the double-exponential function, respectively. The optimal fiber length ℓ_CE, opt_, at which maximum force *F*_max_ can be produced, represents the situation in the fully activated muscle tissue. It is known that in sub-maximally activated fibers the force maximum shifts to longer length with decreasing activity (Rockenfeller and Günther, 2018). The resulting, velocity-dependent fiber force is modeled according to the Hill *relation* (Hill, 1938):

(13) $$\begin{array}{l} F_{\text{CE}}\left(\tilde{a},\ell_{\text{CE}},\frac{\text{d}}{\text{d}t}\ell_{\text{CE}}\right)\\ =\{\begin{array}{ll} F_{\max}\cdot \left(\frac{\tilde{a}\cdot {\tilde{F}}_{\text{isom}}+A_{\text{rel}}}{1-\frac{\text{d}}{\text{d}t}\ell_{\text{CE}}/(B_{\text{rel}}\cdot \ell_{\text{CE},\text{opt}})}-A_{\text{rel}}\right),\; & \text{if}\frac{\text{d}}{\text{d}t}\ell_{\text{CE}}\leq 0\\ F_{\max}\cdot \left(\frac{\tilde{a}\cdot {\tilde{F}}_{\text{isom}}+A_{\text{rel,e}}}{1-\frac{\text{d}}{\text{d}t}\ell_{\text{CE}}/(B_{\text{rel,e}}\cdot \ell_{\text{CE},\text{opt}})}-A_{\text{rel,e}}\right), & \text{if}\frac{\text{d}}{\text{d}t}\ell_{\text{CE}}>0 \end{array}, \end{array}$$

where in the concentric case the Hill parameters *A*_rel_, *B*_rel_ are activity- and length-dependent

(14) $$A_{\text{rel}}(\tilde{a},\ell_{\text{CE}})=\{\begin{array}{ll} A_{\text{rel},0}\cdot (1+3\cdot \tilde{a})/4, & \text{ if }{\tilde{\ell }}_{\text{CE}}\leq 1\\ A_{\text{rel},0}\cdot {\tilde{F}}_{\text{isom}}\cdot (1+3\cdot \tilde{a})/4, & \text{ if }{\tilde{\ell }}_{\text{CE}}>1 \end{array},$$

(15) $$\begin{array}{l} B_{\text{rel}}\left(\tilde{a}\right)=B_{\text{rel,0}}\cdot \left(3+4\cdot \tilde{a}\right)/7. \end{array}$$

In the eccentric case, the Hill hyperbola is reflected to account for experimental findings (Katz, 1939; Komi, 1973; Till et al., 2008):

(16) $$\begin{array}{l} A_{\text{rel,e}}\left(\tilde{a},\ell_{\text{CE}}\right)=-F_{\text{e}}\cdot \tilde{a}\cdot {\tilde{F}}_{\text{isom}},B_{\text{rel,e}}\left(\tilde{a},\ell_{\text{CE}}\right)=\frac{B_{\text{rel}}\cdot \left(1-F_{\text{e}}\right)}{S_{\text{e}}\cdot \left(1+\frac{A_{\text{rel}}}{\tilde{a}\cdot {\tilde{F}}_{\text{isom}}}\right)}. \end{array}$$

The shape parameters *F*_e_, *S*_e_ represent the relative eccentric force limit and the degree of non-differentiability (in terms of a Lipschitz constant) at $\frac{\text{d}}{\text{d}t}\ell_{\text{CE}}=0$ (Haeufle et al., 2014a, Equation 9), respectively.

#### A.0.5. Passive components:

Parallel and serial elastic force-length relations are considered as non-linear and nonlinear-linear elastic springs, respectively:

(17) FPEE(ℓ˜CE)={0, if ℓ˜CE≤LPEE,0FPEE⋅Fmax⋅(ℓ˜CE−LPEE,0ΔWdes+1−LPEE,0)PvEE, if ℓ˜CE>LPEE,0,

(18) $$\begin{array}{l} F_{\text{SEE}}(\ell_{\text{CE}},\ell_{\text{MTU}})\\ =\{\begin{array}{ll} 0, & \text{ if }{\tilde{\ell }}_{\text{SEE}}\leq 1\\ \text{Δ}F_{\text{SEE},0}\cdot {\left(\frac{{\tilde{\ell }}_{\text{SEE}}-1}{\text{Δ}U_{\text{SEE},\text{nll}}}\right)}^{\text{Δ}U_{\text{SEE},\text{nll}}/\text{Δ}U_{\text{SEE},\text{l}}}, & \text{ if }{\tilde{\ell }}_{\text{SEE}}\leq 1+\text{Δ}U_{\text{SEE},\text{nll}}\\ \text{Δ}F_{\text{SEE},0}\cdot \left(1+\frac{{\tilde{\ell }}_{\text{SEE}}-1+\text{Δ}U_{\text{SEE},\text{nll}}}{\text{Δ}U_{\text{SEE},\text{l}}}\right), & \text{ if }{\tilde{\ell }}_{\text{SEE}}>1+\text{Δ}U_{\text{SEE},\text{nll}} \end{array}. \end{array}$$

The PEE slack length as well as characteristic force is given relative to the optimal fiber length and maximum fiber force ($L_{\text{PEE,0}}$ and $F_{\text{PEE}}$), whereas the SEE slack length ℓ_SEE, 0_ and force shape parameter Δ*F*_SEE, 0_ are considered as parameters. The relative SEE length ${\tilde{\ell }}_{\text{SEE}}$ is obtained by ${\tilde{\ell }}_{\text{SEE}}:=\left(\ell_{\text{MTU}}-\ell_{\text{CE}}\right)/\ell_{\text{SEE,0}}$. The slopes of the nonlinear and linear part of the serial elastic FLR are represented by Δ*U*_SEE, nll_ and Δ*U*_SEE, l_, respectively, see also (Günther et al., 2007, Figure 4). The remaining serial damping element is considered to be linearly dependent on MTU force,

(19) $$\begin{array}{l} F_{\text{SDE}}\left(\tilde{a},\ell_{\text{CE}},\frac{\text{d}}{\text{d}t}\ell_{\text{CE}},\frac{\text{d}}{\text{d}t}\ell_{\text{MTU}}\right)=D_{\text{SE}}\cdot \frac{F_{\max}\cdot A_{\text{rel,0}}}{B_{\text{rel,0}}\cdot \ell_{\text{CE,opt}}}\cdot \\ \;\left(\left(1-R_{\text{SE}}\right)\cdot \frac{F_{\text{CE}}+F_{\text{PEE}}}{F_{\max}}+R_{\text{SE}}\right)\cdot \left(\frac{\text{d}}{\text{d}t}\ell_{\text{MTU}}-\frac{\text{d}}{\text{d}t}\ell_{\text{CE}}\right), \end{array}$$

where *D*_SE_ represents the slope and *R*_SE_ the minimum damping coefficient.

#### A.0.6. Contraction dynamics

Solving the force equilibrium (9) with respect to $\frac{\text{d}}{\text{d}t}\ell_{\text{CE}}$ yields a quadratic equation, which can be transferred to a nonlinear ODE

(20) $$\begin{array}{l} \frac{\text{d}}{\text{d}t}\ell_{\text{CE}}=f\left(\tilde{a},\ell_{\text{CE}}\right) \end{array}$$

that is referred to as the term *contraction dynamics*. For details see (Haeufle et al., 2014b, sections 2.5 and 2.6). Note that in the stationary case ($\frac{\text{d}}{\text{d}t}\ell_{\text{CE}}=0$) the MTU force from Equation (9) is given by

(21) $$\begin{array}{l} F_{\text{MTU}}\left(\tilde{a},\ell_{\text{CE}}\right){|}_{\frac{\text{d}}{\text{d}t}\ell_{\text{CE}}=0}=\tilde{a}\cdot F_{\max}\cdot {\tilde{F}}_{\text{isom}}\left(\ell_{\text{CE}}\right)+F_{\text{PEE}}\left(\ell_{\text{CE}}\right). \end{array}$$

### B. ATP reaction kinetics

Basis for our kinetic model of phosphate dynamics is the following reaction scheme (Allen and Orchard, 1987)

(22) $$\begin{array}{l} \text{ATP}<=>\text{ADP}+{\text{P}}_{\text{i}}, \end{array}$$

(23) $$\begin{array}{l} \text{PCr}+\text{ADP}<=>\text{ATP + Cr}, \end{array}$$

(24) $$\begin{array}{l} 2\text{ADP}<=>\text{ATP + AMP}, \end{array}$$

including the auxiliary constraints

(25) $$\begin{array}{l} \text{total adenine concentration:}c_{\text{ad}}=7\text{mM}=\left[\text{ATP}\right]+\left[\text{ADP}\right]\\ \;+\left[\text{AMP}\right], \end{array}$$

(26) $$\begin{array}{l} \text{total creatine concentration:}c_{\text{cr}}=25\text{mM}=\left[\text{PCr}\right]+\left[Cr\right], \end{array}$$

(27) $$\begin{array}{l} \text{total phosphate concentration:}c_{\text{ph}}=46\text{mM}=3\left[\text{ATP}\right]\\ \;+2\left[\text{ADP}\right]+\left[\text{AMP}\right]\\ \;+\left[\text{PCr}\right]+\left[{\text{P}}_{i}\right]. \end{array}$$

For the equilibrium reactions (23) and (24) the law of mass action states

(28) $$K_{\text{ATP}}=\frac{\text{[ATP]}\cdot c_{0}}{\text{[ADP]}\cdot [{\text{P}}_{i}]}=\frac{k_{\text{con}}}{k_{\text{hyd}}}\;,$$

(29) $$\begin{array}{l} K_{\text{crk}}=\frac{\left[\text{ATP}\right]\left[\text{Cr}\right]}{\left[\text{ADP}\right]\left[\text{PCr}\right]}=200, \end{array}$$

(30) $$\begin{array}{l} K_{\text{adk}}=\frac{\left[\text{ATP}\right]\left[\text{AMP}\right]}{{\left[\text{ADP}\right]}^{2}}=1. \end{array}$$

where *k*_hyd_ and *k*_con_ denote the ATP hydrolysis and condensation rate constant, respectively. The equilibrium constants for creatine kinase (crk) and adenylate kinase (adk) are likewise taken from Allen and Orchard (1987). It should be noted that there exist other sources with different values e.g., for initial [PCr] (Allen et al., 2008; Karatzaferi et al., 2001), as well as the equilibrium constants for the creatine kinase (Bechtel and Best, 1985; Gadian et al., 1981; Lawson and Veech, 1979; Sahlin et al., 1975) and the adenylate kinase (Kübler and Katz, 1977; Linari et al., 2010; Newsholme, 1 72; Noda, 1973) reaction. For the sake of consistency, however, we obtained all values from the same source, because the presented data do not allow a detailed assessment and thus estimation of these parameters.

For the pseudo-first order (i.e., the concentration of water is assumed to remain constant) reaction [Equations (22) or (1) together with (28)] it holds that

(31) $$(-\frac{\text{d}}{\text{d}t}[\text{ATP}]\;(t)\;=\frac{\text{d}}{\text{d}t}[\text{ADP}]\;(t)=\frac{\text{d}}{\text{d}t}[P_{i}](t)$$

(32) $$=k_{\text{hyd}}\left(q,{\tilde{\ell }}_{\text{CE}}\right)\cdot [\text{ATP}]\cdot c_{0}-k_{\text{con}}\cdot [\text{ADP}]\cdot [{\text{P}}_{i\prime }]$$

where [*ATP*](*t*) and [P_*i*_](*t*) denote the time evolution of [ATP] and [P_*i*_]. Note that *k*_hyd_ is assumed to depend on the current amount of calcium-bound troponin *q* and the current CE length ${\tilde{\ell }}_{\text{CE}}$, cf. section 3. As an initial condition, set [*ATP*](0) = [*ATP*]_0_. As the equilibrium constitutes for a rather lumped description of the Lymn-Taylor cycle (see Discussion 5.2), the choice of a suitable value for *k*_con_ constitutes for the limiting factor of the reaction, approximately 0.1 Hz (Linari et al., 2010).

In order to obtain a numerical solution for [*ATP*](*t*), plug Equation (25) in Equations (27) and (30) as well as Equation (26) in Equation (29), yielding

(33) $$\begin{array}{l} \left[\text{ADP}\right]=\frac{-\left[\text{ATP}\right]+\sqrt{4\cdot K_{\text{adk}}\cdot c_{\text{ad}}\cdot \left[\text{ATP}\right]-\left(4\cdot K_{\text{adk}}-1\right)\cdot {\left[\text{ATP}\right]}^{2}}}{2\cdot K_{\text{adk}}}, \end{array}$$

(34) $$\begin{array}{l} \left[\text{AMP}\right]=c_{\text{ad}}-\left[\text{ATP}\right]-\left[\text{ADP}\right], \end{array}$$

(35) $$\begin{array}{l} \left[\text{PCr}\right]=\frac{c_{\text{cr}}\cdot \left[\text{ATP}\right]}{\left[\text{ATP}\right]+K_{\text{crk}}\cdot \left[\text{ADP}\right]}, \end{array}$$

(36) $$\begin{array}{l} \left[\text{Cr}\right]=c_{\text{cr}}-\left[\text{PCr}\right]. \end{array}$$

The hereof obtained expressions for [*PCr*] (Equation 35) and [*ADP*] (Equation 33) are plugged in Equation (27), resulting in variable [*ATP*]:

(37) $$0=c_{\text{ad}}-c_{\text{ph}}+[{\text{P}}_{i}]+2\cdot [\text{ATP}]+[\text{ADP}]+[\text{PCr}],$$

thus expressing [*ATP*] as a function of the only remaining variable [P_*i*_]. In fact, both Equations (32) and (37) imply the same property of the reaction scheme (22)-(30), namely, that [*ATP*], [*ATP*] or [P_*i*_] could either be treated as a new state variable: if one is known, the others can be calculated. Here, the root [*ATP*]([P_*i*_]) of Equation (37), with additionally using Equations (33) and (35), feeds the right side of Equation (32), which enables to continuously update [P_*i*_] by integrating the ODE. For these calculations, self-implemented Runge-Kutta and bisection methods were used. Initial conditions of all reactants followed from assuming [*ADP*]_0_ = 0.01 mM (Allen et al., 2008) in the resting fiber: [*ATP*]_0_ = 6.99 mM, ${\left[AMP\right]}_{0}=10^{-5}\text{mM}$, [*PCr*]_0_ = 19.45 mM, [*Cr*]_0_ = 5.55 mM , [P_*i*_]_0_ = [*Cr*]_0_ + [*ADP*]_0_ = 5.56 mM. Further from physiological experiments, a minimum of remaining ATP, namely [*ATP*]_min_ = 1.2 mM was found in exhausted fibers (Allen and Orchard, 1987) and included as a constraint in the bisection method. Resulting time courses of most involved reactants, including their length-dependency, are displayed in Figure A1 in Supplementary Material.

The addressed interaction between ATP, ADP, and P_*i*_ is known to influence the *chemical potential* (Zhang and Feng, 2016) or *affinity* (Allen and Orchard, 1987; Cooke, 2007; Hancock et al., 2005) of ATP, write μ_ATP_, which constitutes for the new state variable of phosphate dynamics. In a nutshell, the position of equilibrium (22) influences the amount of free Gibbs energy from ATP hydrolysis via

(38) $$\mu_{\text{ATP}}:=-\text{Δ}G_{\text{ATP}}^{{}^{\circ}}+R\cdot T\cdot \ln\left(\frac{[\text{ATP}]\cdot c_{0}}{\left[\text{ADP}]\cdot [{\text{P}}_{i}\right]}\right),$$

where $\Delta G_{\text{ATP}}^{{}^{\circ}}\approx -30$ kJ/mol (Allen and Orchard, 1987; Cooke, 2007; Guynn and Veech, 1973; Rosing and Slater, 1972; Zhang and Feng, 2016) denotes the standard free enthalpy, *R* = 8.31·10^−3^ kJ/(mol·K) the ideal gas constant, *T* = 303 K a temperature of 30°C, and *c*_0_ = 1 M the standard concentration. The latter serves as a normalization factor. An estimate for the theoretically maximum value for the chemical potential in the resting fiber can be given by

(39) $$\mu_{\text{ATP},\text{max}}=-\text{Δ}G_{\text{ATP}}^{{}^{\circ}}+R\cdot T\cdot \ln\left(\frac{{\left[\text{ATP}\right]}_{0}\cdot c_{0}}{{\left[\text{ADP}\right]}_{0}\cdot {\left[P_{i}\right]}_{0}}\right)\approx 59.6\frac{\text{kJ}}{\text{mol}}$$

in accordance with literature data (Barclay, 2015). The relative (normalized) ATP affinity is then defined as ${\tilde{\mu }}_{\text{ATP}}:=\mu_{\text{ATP}}/\mu_{\text{ATP,max}}$, for which a time course is likewise displayed in Figure A1 in Supplementary Material.

The time derivative of ${\tilde{\mu }}_{\text{ATP}}$ as used in Equation (8) can finally be calculated as

(40) $$\frac{\text{d}}{\text{d}t}{\tilde{\mu }}_{\text{ATP}}=\frac{R\cdot T}{\mu_{\text{ATP},\text{max}}}\cdot \left(\frac{\frac{\text{d}}{\text{d}t}[\text{ATP}]}{\left[\text{ATP}\right]}-\frac{\frac{\text{d}}{\text{d}t}[\text{ADP}]}{\left[\text{ADP}\right]}-\frac{\frac{\text{d}}{\text{d}t}[P_{i}]}{\left[{\text{P}}_{i}\right]}\right)\sim -k_{\text{hyd}},$$

where the latter proportionality holds after inserting Equation (32) in Equation (40) and assuming a constant concentration of the involved phosphate compounds.

### C. Parameter estimation and sensitivity analysis

For the mathematical formalization of the introduced model, define the domain of the neural stimulation *u* = *u*(*t*) as $U:=C^{pw}\left(\left[t_{start},t_{end}\right],\left[0,1\right]\right)$, i.e., the set of piece-wise continuous functions mapping the experimentally covered time horizon [*t*_*start*_, *t*_*end*_] (here: *t*_*start*_ = −0.1s and *t*_*end*_ = 1.1s) to the interval [0, 1]. Let $\Lambda_{M}\in {\text{R}}^{d}$ denote the tuple of model parameters, representing the corresponding muscle *M*∈{GAS, PLA}. Denote the modeled time evolution of muscle force by

(41) $$\begin{array}{l} F_{M}:U\times {\text{R}}^{d}\to C\left(\left[t_{start},t_{end}\right],\text{R}\right),\;F_{M}\left(u,\Lambda_{M}\right)=F_{M}\left(t\right). \end{array}$$

Our aim is to find the optimal parameter set $\Lambda_{M}^{*}$ such that for a given experimental stimulation protocol (input) $\bar{u}$ the measured force (data) ${\bar{F}}_{M}\left(t\right)$ is at best approximated by the model force (output) *F*_*M*_(*t*) in a least-squares sense:

(42) $$\begin{array}{l} \Lambda_{M}^{*}=\underset{\Lambda_{M}\in {\text{R}}^{d}}{arg min}J\left(\Lambda_{M}\right):=||F_{M}\left(\bar{u},\Lambda_{M}\right)-{\bar{F}}_{M}\left(t\right){||}_{2}. \end{array}$$

For solving problem (42), we used a pre-implemented MatLab (R2018b, The MathWorks, Natick, USA) routine, namely *lsqcurvefit*, utilizing a trust-region-reflective algorithm.

In order to asses the influence of every model parameter at each time instance, additional sensitivity values are provided in section 4.2. The local sensitivity (Rockenfeller et al., 2015; Saltelli et al., 2000) of a certain parameter λ ∈ Λ_*M*_ is thereby defined as the partial derivative of the objective function with respect to λ at the optimum, i.e., $S_{M,\lambda }\left(t\right):=\partial J\left(\Lambda_{M}^{*}\right)/\partial \lambda$. A large positive (negative) sensitivity means that only relatively small changes in λ to larger (smaller) values are needed in order to attune model output and measured data. The smaller the sensitivity the larger the required relative change. Hence, parameters with high absolute sensitivities have to be treated with high accuracy, but are easier determinable than insensitive parameters. Conversely, the model is not prone to uncertainties in parameters with comparatively low absolute sensitivities, but these parameters are hardly determinable, cf. (Rockenfeller, 2016, Ch. 5.3) and (Rockenfeller and Günther, 2016).

### List of symbols and abbreviations

**Table:**

**Abbreviation**	**Meaning**
ADP	Adenosine diphosphate
AMP	Adenosine monophosphate
ATP	Adenosine triphosphate
Ca (Ca^2+^)	Calcium (ion)
CE	Contractile element
Cr	Creatine
GAS	*M. gastrocnemius*
MTU	Muscle tendon unit
ODE	Ordinary differential equation
P_*i*_	Inorganic phosphate
PCr	Creatine phosphate
PEE	Parallel elastic element
PLA	*M. plantaris*
pH	Power/potential of hydrogen
SDE	Serial damping element
SEE	Serial elastic element

**Table:**

**Symbol**	**Value**	**Unit**	**Meaning**
ã	$\tilde{q}\cdot {\tilde{\mu }}_{\text{ATP}}$	[]	Activity
*A*_rel, 0_	Parameter	[]	Hill parameter
*B*_rel, 0_	Parameter	[1/s]	Hill parameter
*D*_SDE_	Parameter	[]	Shape parameter of the SDE force-force curve
Δ*F*_SEE, 0_	Parameter	[N]	Shape parameter of the SEE force-length curve
Δ*U*_SEE, nll_	Parameter	[]	Shape parameter of the SEE force-length curve
Δ*U*_SEE, l_	Parameter	[]	Shape parameter of the SEE force-length curve
Δ*W*_asc_	Parameter	[]	Shape parameter of the isometric force-length relation
Δ*W*_des_	Parameter	[]	Shape parameter of the isometric force-length relation
*F*, *F*_X_	Free	[N]	Force, force of element X
*F*_e_	Parameter	[]	Shape parameter of the eccentric force-velocity branch
${\tilde{F}}_{\text{isom}}\left(\ell_{\text{CE}}\right)$	Length-dependent	[]	Normalized isometric force-length relation
*F*_max_	Parameter	[N]	Maximum isometric force at ℓ_CE, opt_
$F_{\text{PEE}}$	Parameter	[]	Shape parameter of the PEE force-length curve
Δ*G*°	Reaction-dependent	[kJ/mol]	Standard-state free (Gibbs-)energy of reaction
*K*	Free or state-dependent	[]	Equilibrium constant
*k*	Free or state-dependent	[1/s]	(Chemical) reaction rate constants
${\hat{k}}_{\text{hyd}}$	Parameter	[1/s]	ATP hydrolysis rate constant
ℓ_CE_	State	[m]	Length of the contractile element
ℓ_CE, opt_	Parameter	[m]	Optimal length of the contractile element
${\tilde{\ell }}_{\text{CE}}$	ℓ_CE_/ℓ_CE, opt_	[]	Relative length of the contractile element
$L_{\text{PEE}}$	Parameter	[]	Shape parameter of the PEE force-length curve
ℓ_SEE, 0_	Parameter	[m]	Slack length of the serial elastic element
M, mM, μM	10^0^, 10^−3^, 10^−6^	[mol/l]	Molar, millimolar, micromolar
*m*	Parameter	[1/s]	Time constant (Hatze)
μ_ATP_, μ_ATP, max_	State-dependent	[kJ/mol]	(maximum) chemical potential of ATP
${\tilde{\mu }}_{\text{ATP}}$	μ_ATP_/μ_ATP, max_	[]	Relative chemical potential of ATP
ν	Parameter	[]	Exponent (Hatze)
ν_asc_	Parameter	[]	Shape parameter of the isometric force-length relation
ν_des_	Parameter	[]	Shape parameter of the isometric force-length relation
ν_PEE_	Parameter	[]	Shape parameter of the PEE force-length curve
$\tilde{q}$	${\tilde{q}}_{\text{min}}\leq \tilde{q}\leq 1$	[]	Troponin-activity (Hatze)
${\tilde{q}}_{\text{min}}$	≤ 0.01	[]	Minimum troponin-activity
*R*_SDE_	Parameter	[]	Shape parameter of the SDE force-force curve
*S*_e_	Parameter	[]	Shape parameter of the eccentric force-velocity branch
$\varpi \left({\tilde{\ell }}_{\text{CE}}\right)$	$\varpi_{c}\cdot {\tilde{\ell }}_{\text{CE}}$	[]	Length-dependent volumetric troponin C density
ϖ_opt_	Parameter	[]	Constant (Hatze)
*u*	0 ≤ *u*(*t*) ≤ 1	[]	Neural stimulation
[X]	Free	[mol/l]	Molar concentration of substance X
[X]_0_	Free	[mol/l]	Initial molar concentration of substance X
[X]_min_	Free	[mol/l]	Minimum concentration of X
